# Conversational System as Assistant Tool in Reminiscence Therapy for People with Early-Stage of Alzheimer’s

**DOI:** 10.3390/healthcare9081036

**Published:** 2021-08-12

**Authors:** Victor Morales-de-Jesús, Helena Gómez-Adorno, María Somodevilla-García, Darnes Vilariño

**Affiliations:** 1 Facultad de Ciencias de la Computación, Benemérita Universidad Autónoma de Puebla, Avenida San Claudio y 14 Sur, Ciudad Universitaria, Puebla 72570, Mexico; victor.morales@cs.buap.mx (V.M.-d.-J.); darnes@cs.buap.mx (D.V.); 2 Instituto de Investigaciones en Matemáticas Aplicadas y en Sistemas, Universidad Nacional Autónoma de México, Circuito Escolar 3000, C.U., Coyoacán, Ciudad de México 04510, Mexico; helena.gomez@iimas.unam.mx

**Keywords:** conversational system, Alzheimer’s disease, reminiscence therapy, mental health

## Abstract

Reminiscence therapy is a non-pharmacological intervention that helps mitigate unstable psychological and emotional states in patients with Alzheimer’s disease, where past experiences are evoked through conversations between the patients and their caregivers, stimulating autobiographical episodic memory. It is highly recommended that people with Alzheimer regularly receive this type of therapy. In this paper, we describe the development of a conversational system that can be used as a tool to provide reminiscence therapy to people with Alzheimer’s disease. The system has the ability to personalize the therapy according to the patients information related to their preferences, life history and lifestyle. An evaluation conducted with eleven people related to patient care (caregiver = 9, geriatric doctor = 1, care center assistant = 1) shows that the system is capable of carrying out a reminiscence therapy according to the patient information in a successful manner.

## 1. Introduction

Dementia is a neurodegenerative and progressive condition that is characterized by the alteration of cognitive processes, behavior, emotional state and the limitation in the ability to develop activities of daily living [1]. The most common type of dementia is Alzheimer’s disease (AD), covering up to 80% of cases. It is also one of the main reasons for disability in elderly people over 65 years, generating dependence on those who suffer from it. In 2019, there were over 50 million people living with dementia globally, and it is estimated that by 2050 this quantity will increase up to 152 million [2].

According to this trend, an accelerated growth of the elderly population in Mexico is expected to increase by 300% in the next 20 years [3]. The analysis of these data is relevant as it shows a need in the development and implementation of the necessary care for the elderly population group. Almost a third part of this population will be older adults with various levels of dependence, and as mentioned above, the main cause of dependence in the elderly is precisely dementia.

Although dementia is currently not curable, people having this condition can preserve a good quality of life as long as possible if properly managed. In this context, there are non-pharmacological treatments focused on mitigating the psychological, behavioral and cognitive impairment symptoms. It is preferable that the treatment of a patient with Alzheimer’s disease (PwAD) begins with a non-pharmacological intervention, since this type of treatment promotes the use of different methods and techniques to provide emotional and physical stability to PwADs without the side effects of pharmacological treatments [4,5]. The benefits of this type of interventions can be maximized through the implementation of appropriate environments, stimulating tasks and a diverse kind of therapies according to the needs of each PwAD [6].

Reminiscence therapy (RT) is a kind of such interventions. RT is based on conversations between the PwAD and a therapist, a health specialist or his/her caregiver. These conversations are guided by the therapist and focusing on activities and experiences about the past. In the RT the PwAD can be guided chronologically through his/her life history if the aim is to perform an analysis and review of the patient’s life. But it is also possible to evoke specific pleasant memories concerning the patient’s relevant experiences according to RT purpose [7]. This type of therapy focuses on the long-term memory of the PwADs using personal information that is strongly related to them and relatively easy to remember. In the early stage of AD the short-term memory is mainly affected whereas the long-term memory is still well preserved [8]. The benefits of subjecting PwADs to RT have been analyzed in several studies and it has been observed that carrying it out constantly supports improving unstable psychological and emotional states, as well as increasing their sociability and trust [9,10,11].

Nevertheless, it is difficult for most PwADs to consult a health specialist or therapist periodically, so this task is assumed by their caregivers, who are usually a family member. Moreover, due to the overburden of the caregivers, they are quite limited in the time they can use to provide the RT. Therefore, it is necessary to design and implement technology that can support the treatment of patients on a regular basis and maximize the benefits that this offers.

In this sense, to our knowledge there is no a conversational system that is aimed on providing RT in the Spanish language on an individualized basis to PwADs. Although there are studies focused on the dementia domain, most of them are focused on trying to make an early diagnosis of dementia and the development of cognitive and emotional therapy assistants. In this regard, the objective of this work is focused on the creation of a conversational system capable of generating personalized conversations to support caregivers and therapists to provide RT in a constant and personalized manner to PwADs.

This paper has the following structure: Section 2 provides a brief overview of related proposals working on the health care and the dementia domain in particular. Section 3 describes the conversational system proposed in this work and the methods to gather the information from the PwADs and how this information is used by the system to personalize the reminiscence therapy. Section 4 presents the evaluation scheme followed to assess the appropriateness of the system to provide reminiscence therapy and the results are reported in Section 5. We discuss about the results obtained in Section 6. Finally, some conclusions and future work are included in Section 7.

## 2. Related Work

Nowadays, there is a great interest in the development of dialogue systems, conversational systems, and interactive systems focused on health care [12,13,14] and mental health [15,16,17].

In the dementia domain these systems are mainly focused on providing support to caregivers and PwADs covering a diverse range of applications. Ranging from those focused on early detection and diagnosis, the development of cognitive assistants, up to those focused on supporting psychological and emotional therapy to PwADs. Some relevant research studies in each of the previously specified approaches are described in this section. Table 1 summarizes these proposals.

### 2.1. Systems Focusing on Diagnosis

Studies working on detection and diagnosis of dementia implement methods to classify symptoms and identify distinctive characteristics for early detection. On this line, Tanaka et al. [18] propose a virtual avatar with spoken dialogue functionalities, which conducts an interview with patients, the questions are based on the Mini-Mental State Examination (MMSE) [19], the Wechsler Memory Scale (WMS) [20] and other neuropsychological tests. The authors record the interaction between the avatar and the patient, extract diverse audiovisual features and subsequently implement classification algorithms. They claim to achieve an accuracy of 0.93 in the detection of dementia.

Another approach used on diagnosis of dementia is proposed by Chinaei et al. [21]. They analyze some linguistic characteristics which are considered as verbal indicators of confusion in people with dementia, these are: the richness in the vocabulary, the analysis of the structure of the syntactic tree of the sentences and acoustic signals. Finally, they apply machine learning algorithms to identify confusion within the dialogue, reaching 82% of accuracy.

### 2.2. Systems Focusing on Cognitive Support and Therapy

Some proposals focus on the development of assistive technology to help people with dementia to carry out their daily life activities, as well as to support different type of therapy for mitigating psychological, emotional and, behavioral symptoms.

In this sense, Yasuda et al. [22] implemented a system that provides occasional reminders and reminiscence conversations remotely. Through video calls they aim to improve psychological stability and to assist people with dementia to perform simple tasks. They observed that after the intervention, the psychological stability of a PwAD persisted for up to three hours after the conversation and the success rate when completing a task was increased to 80%. This type of proposal is compared in more detail against other instruction monitoring strategies by Perilli et al. [23].

Rudzicz et al. [24] describes the development of a mobile robot called ED, which supports people with dementia in the performance of tasks through visual monitoring and verbal prompts. They analyze the voice interactions between ED and each of the patients involved in the study (*n* = 10). Their analysis reveals that for patients who have high levels of confusion, it is very likely that they ignore the robot at the time it provides assistance to perform a task, which represent 40% of the cases of the trial group under study.

Similarly, Wolters et al. [25] analyzed the interaction between patients with dementia and a simulated intelligent cognitive assistant supporting patients to develop their daily activities. The assistant can detect when there is a problem to perform the task and then offers assistance based on the context of the moment of confusion. The authors assess the performance of the system in three focus groups: patients with dementia, their caregivers, and older people without a diagnosis of dementia. Finally, the analysis of the data obtained from each of these groups showed that the interaction style and the type of voice have a greater relevance for the patients. They also found that it is desirable a personalization in the interaction that exists between the cognitive assistant and the patient.

A study conducted by Casey et al. [26] describes how the perceptions of people with dementia and other key stakeholders are helpful to develop a companion robot following a user led design approach. In their research, they identify elements of importance for patients, which make a companion robot have a higher level of acceptance. The authors mention that some relevant aspects to be implemented into the robot described by the patients and health care staff are: to have a friendly face; to speak slowly and loudly; to remind them when to take their medications and the need for the robot to know interests, preferences and the patient’s life history and use this information to motivate the conversation and reminiscent about events that patients can still remember.

#### Systems Focusing on Therapy in Spanish Language

It is worth noting that most of the studies on developing conversational and interactive systems for the dementia domain focus on English language. But since the system described in this work is for the Spanish language, it is relevant to mention the few similar studies developed on this language.

Navarro et al. [27] designed an assisted cognition system and the results on the use and adoption of the system are analyzed in two branches: (1) to support in providing occupational therapy and (2) the effectiveness of the system to mitigate behavioral changes in PwADs. They carried out an evaluation of the system with two couples (PwAD-caregiver) and observed that the personalization of the intervention and the tactile interface for interaction facilitate the adoption of the system. They also analyzed that implementing this type of systems in the treatment of PwADs reduces the caregivers burnout.

Another relevant study was conducted by Cruz-Sandoval et al. [28], a social robot (Eva) was implemented as facilitator of a cognitive stimulation therapy in a nursing home on a nine-week study. During the intervention the robot implements direct interactions with PwADs through cognitive games, reminiscence, and music therapy. After conducting a evaluation with a group of eight PwADs, they observed a statistically significant decrease in the dementia-related behavioral symptoms and positive short-term effects after the each session, providing evidence that this type of approaches could improve the quality of life of people with dementia.

## 3. Materials and Methods

### 3.1. Design of the Conversational System

**Figure 1:**
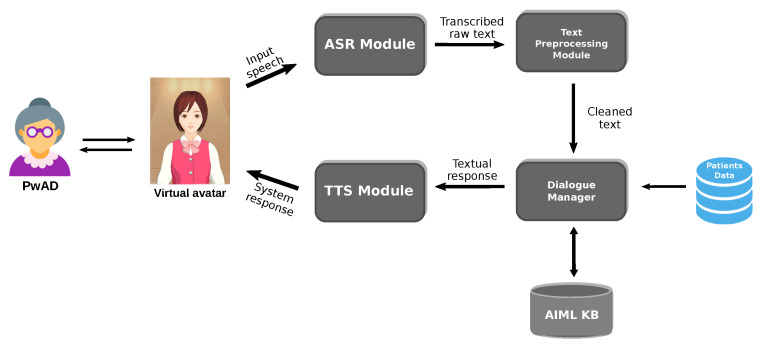
Architecture of the conversational system.

The architecture of the conversational system is shown in Figure 1. The system was developed following a modular architecture design that allows us separating the different tasks to be performed by the system, which will facilitate its maintenance, scalability, and improvements in future versions.

The modules that integrate the conversational system are as follows: the Automatic Speech Recognition (ASR) Module, the Text Preprocessing Module, the Dialogue Manager, the AIML-based Knowledge Base, the Text-to-Speech (TTS) Module, a database containing the data from the PwADs used to personalized the dialogues, and a virtual avatar who serves as the entity interacting with the PwADs.

#### 3.1.1. ASR Module and TTS Module

The ASR Module is where the input speech of the user (PwAD) is recognized and transcribed into text. In this regard, the Google Cloud Speech-to-Text service (https://cloud.google.com/speech-to-text/, accessed on 10 March 2021) was implemented because of according some performed experiments, this service shows a better performance to recognize the utterances of older-people than other similar services. The results of the these experiments are described in Section 5.1.

On the other hand, the TTS module where the response of the system is synthesized in speech to the user, the IBM Watson Text-to-Speech service (https://www.ibm.com/cloud/watson-text-to-speech, accessed on 10 March 2021) was implemented, because it offers a natural-sounding speech in Spanish language using a Latin American dialect and controllable speech attributes. According to some dialogue strategies to improve the communication with PwADs described in Section 3.1.4, the default behavior of the TTS service was configured to have a speed of 0.8 respect to the normal speech speed, in order for the TTS module to produce a clear and slow speech which it is recommended to be used in communication with people with dementia.

#### 3.1.2. Text Preprocessing Module

This module cleans and normalizes the text transcribed by the ASR module to eliminate text features that will not be needed later or may even affect the performance of the system. The tasks performed within this module are: removing punctuation marks and non-alphanumeric characters, converting all characters to lower case and removing consecutively repeated words, as it was observed that this features is often due to speech recognizer deficiency to deal with false start errors in spontaneous speech [29] (e.g., este este, um um, ee ee). The tasks performed in this module subsequently allow a better comparison between the user utterances and the patterns defined in the AIML knowledge base.

#### 3.1.3. Dialogue Manager

The Dialogue Manager (DM) is in charge of handling the conversation shifts between the user and the system, determining the actions to perform during the interaction according to the current state of the dialogue to determine which will be the most appropriate response. In this sense, the DM manages the information obtained from the database and how this information is used in the AIML knowledge base to select the response based on the utterance received from the PwAD. The selected response can be a generic response or a customized response using PwAD information according to the dialog context as described in Section 3.3.

#### 3.1.4. AIML-Based Knowledge Base

A set of dialogue scenarios or contexts has been created using the AIML language to define them. The delimitation of these dialogue contexts is based on the topics on which the conversational system is capable of conducting the RT, as described in Section 3.1 these are: personal information, family relationships, and life history and lifestyle. To date, a total of 20 dialogue contexts have been established. In Table 2 a description of each one is given.

As can be observed, most of the dialogue contexts are focused on the PwAD’s interests, so it is essential to know this information about each PwAD in order to be able to personalize the dialogue on a case-by-case basis. The way to collect this information from the PwAD is described in Section 3.2.

Within the knowledge base created, there are two types of dialogue templates: generic dialogue templates and customized dialogue templates. Generic dialogue templates are used in dialogue contexts where dialogue personalization is not strictly necessary. On the other hand, customized dialogue templates are used in most contexts because what is pursued is precisely the personalization of the conversation according to the PwAD’s interests. A more detailed description of dialogue personalization is described in Section 3.3.

In the majority of the customized dialogue templates, more than one response is defined and can be selected by the system so that the conversation does not become repetitive. In addition, at each turn or system response, the PwAD is encouraged to continue the conversation by asking a new question in relation to the context of the current conversation.

It is worth noting that for the design and creation of the dialogue templates in each context, we implemented the recommendations suggested in the literature to establish effective communication strategies when interacting with people with dementia [30,31,32,33]. Some of these strategies consider: the use of simple and short sentences, personalizing the conversation, talking about the PwAD’s topics of interest, using praise and positive reinforcement phrases. The implementation of these strategies in interaction systems focused on people with dementia has shown positive results [34].

#### 3.1.5. Virtual Avatar

Because PwADs have shown a better acceptance when interacting with interactive systems that have some type of embodiment (robots, avatars, humanoids) compared to those that only use voice or text as the communication interface [35]. In this work we have considered the implementation of a virtual avatar that will function as a communication interface between the user and the conversational system.

To implement the virtual avatar, the MMDAgent toolkit [36] is used. This free software tool offers the possibility to build voice interaction systems. A main reason for selecting this tool lies in the motor characteristics of the avatar. It is possible to configure arm and head movements and facial expressions according to the type of conversation.

### 3.2. Gathering Data from PwADs

Since the conversational system is intended to generate personalized conversations for each PwAD, it is necessary to have relevant information about each PwAD. Therefore, a medically validated form [37] is used as basis for determining what kind of information is relevant and needs to be gathered people with dementia in care centers to provide better care.

The questions to recollect this data are classified in three different topics: (1) personal information, (2) family relationships, and (3) life history and lifestyle of the PwAD. The guidelines established in the used forms recommend that it is preferable that the information must be provided by the caregiver or a PwAD’s close relative whenever possible.

In order to gather this information, a web application has been developed that allows to enter the information through a set of forms according to the three above mentioned topics. A set of 32 questions have been selected and distributed as follows: 11 questions referring to personal information, 4 questions referring to family relationships and 17 questions referring to life history and lifestyle (e.g., preferences, habits and hobbies). All these questions are related to the contexts described in Table 2.

**Figure 2:**
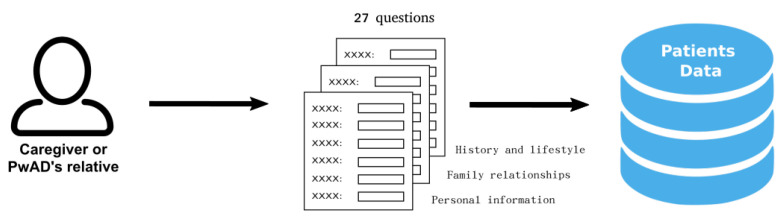
Gathering data about the PwAD, the stored information is about personal information, family relationships, life’s history and lifestyle.

Once the forms are completed by the caregiver or PwAD’s relative, this information is stored in a database as is shown in Figure 2. The database stores the information about all PwADs and is consulted by the Dialogue Manager to create a profile for every single PwAD with the aim to personalize the conversation between the system and the PwAD according to the information collected.

### 3.3. Personalizing the Dialogue

The main objective of the conversational system is to provide reminiscence therapy to PwADs. For this purpose, it is essential that the conversation carried out by the system should be personalized for each PwAD. In this sense, the personalization that the system offers to the user is based on the information collected and stored in the database to be used in a set of dialogue templates according to the different dialogue contexts which were described in Section 3.1.4.

**Figure 3:**
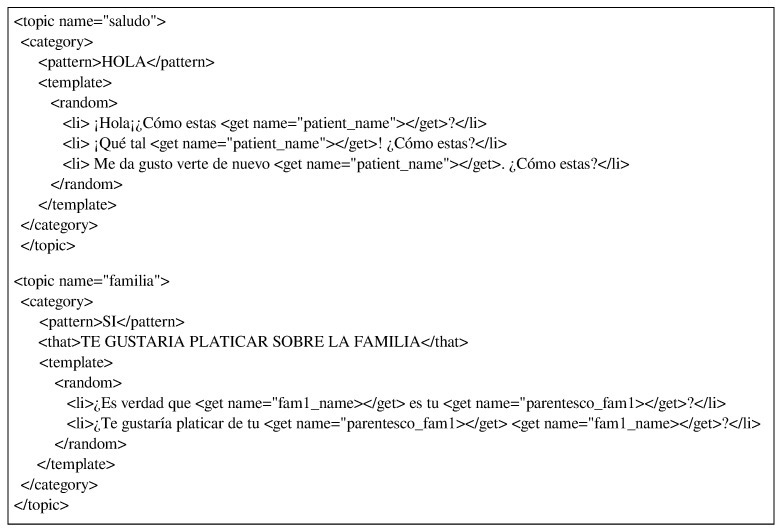
Personalized dialogue template using the PwAD information.

Figure 3 shows two instances of the dialogue templates created to integrate PwAD information into the dialogues according to the scenario or context of conversation.

In the first dialogue template (upper), the topic or dialogue context is regarding to greeting (saludo) and the template use the information retrieved from the database referring only to the PwAD’s name (*“patient_name”*) in this case.

In the second dialogue template (lower), the topic of the dialogue is family (familia) and the information used is the name of the first family member registered for that PwAD (*“fam1_name”*) and their family relationship with the PwAD (*“parentesco_fam1”*). Thus, if the retrieved values were *fam1_name=“Patricia”* and *parentesco_fam1=“hija” (daughter)*, then the posible answers by the system would be: *¿Es verdad que Patricia es tu hija?* (*Is it true that Patricia is your daughter?*) or *¿Te gustaría platicar de tu hija Patricia?* (*Would you like to talk about your daughter Patricia?*).

In the above examples only information about PwAD’s name and a relative is used. However, all PwAD-related information is retrieved from the database and provided by the Dialog Manager to the AIML knowledge base because there are several dialogue templates in which all the collected information is used.

## 4. Evaluation

At this stage of the work, the evaluation of the conversational system has focused on determining how appropriate the responses provided by the system are, in order to carry out reminiscence therapy adequately in a PwADs. This, with the intention that the system can be initially evaluated by caregivers, PwAD’s relatives or people related to PwAD care and based on their observations, subsequently evaluate the conversational system interacting with real PwADs.

A static context evaluation scheme has been followed [38] for evaluating the appropriateness of the responses given by the system to the user utterances. This scheme suggests that in order to facilitate the participation of the evaluators of the system, they take the role of bystander. This mean that the evaluator does not actively participate in the conversation and more than one evaluator can judge a dialogue for appropriateness of response.

Thus, the evaluator does not interact directly with the system but is provided with a set of dialogues carried out between the system and a user. Each of these dialogues is composed by a dialogue context (at least 4 turns of interaction between the system and the user) and a set of candidate responses, one of them is the response provided by the system. For each of the dialogues being evaluated, the evaluator must select the response that they consider the system should give according to the actual dialogue context.

### Setup and Procedure

To conduct the evaluation, a set of 40 text-based dialogues between the system and a user (simulated PwAD) was generated. The PwAD’s information used for the personalization of the therapy is also simulated since it was not taken from any real PwAD yet. The generated dialogues are based on the topics over which the system has the ability to converse (described in Table 2), generating two dialogues per topic.

**Figure 4:**
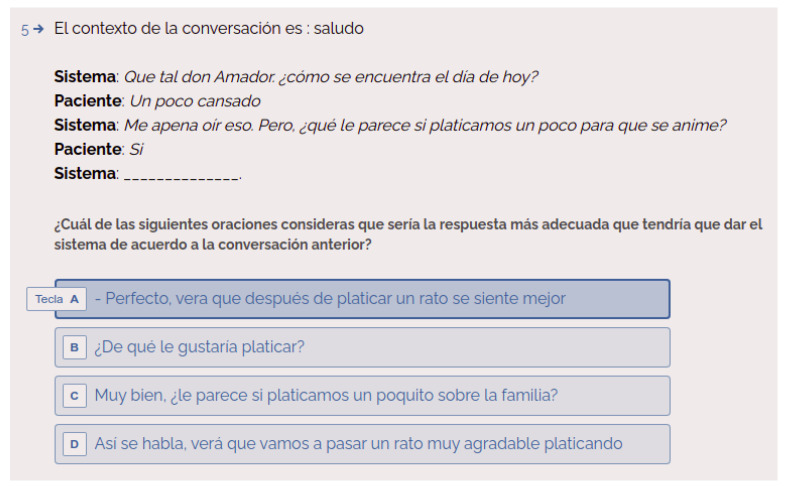
Example of a dialogue where the evaluator has to select the most appropriate response in accordance with the dialogue that is taking place.

In order to make the set of generated dialogues available to the evaluators, a web form was created through which the evaluators had access to the set of dialogues (https://ylcnhgpssgf.typeform.com/to/BZcWGb6V, accessed on 30 March 2021). Figure 4 shows a capture of a dialogue and the candidate responses to follow up the conversation.

In the dialogue showed in Figure 4, the evaluator has to vote (select) for the option that they consider is the most appropriate response respect to the dialogue that is being held. This has to be done for each of the 40 dialogues and its candidate responses. The set of dialogues was evaluated by a total of 11 people. Thus, each evaluator judge the complete set of dialogues.

The method for recruitment of the evaluators was through a call for participation posted on social networks in groups related to providing information and support to PwADs’ relatives or caregivers. From the people who expressed interest in participating, those who met the following selection criteria were selected: (1) Have a PwAD under their direct care and belong to one of the following categories: therapist, geriatric doctor, care center assistant or primary caregiver, and (2) Being familiar with reminiscence therapy and at least once having provided it. From 18 people who expressed interest in participating, 11 people were selected who met the selection criteria. Thus, the participants were: nine caregivers, one geriatric doctor and one care center assistant. None of the participants received any type of economic compensation for their participation. The set of evaluations is freely available (https://github.com/vicmman/cs_evaluation, accessed on 5 April 2021).

Once the evaluation process is concluded, a rating of votes is obtained for each of the candidate responses within each dialogue. Thus, a evaluation based in the Voted Appropriateness metric proposed by Gandhe et al. [38] is performed, where the value $R_{voted}=V\left(u_{t},context_{t}\right)$ is calculated for each response. The $R_{voted}$ value represents the number of evaluators who selected (vote) the utterance $u_{t}$ as an appropriate response in the context $context_{t}$ of the evaluated dialogue *t*. In the next section, the results of this evaluations are presented.

This evaluation scheme also allows us to identify if according to the evaluator criteria the overall system performance is adequate and therefore it is suitable to carry out the reminiscence therapy by implementing the conversational system proposed in this work on real PwADs.

## 5. Results

This section initially describes the results obtained from the benchmarking of the ASR systems. Subsequently, the results of the evaluation of the conversational system are presented.

### 5.1. Results of Experiments on ASR Systems

It is important to determine which ASR system offers better performance in this task, since the error rate of the ASR will affect the behavior of the conversational system. A comparative evaluation of different ASR systems has been carried out, considering only those that claim to achieve an accuracy above 85% in Spanish language recognition.

The ASR systems analyzed in this work were Google Speech-to-Text (ASR-1), IBM Watson Speech to Text (ASR-2) and Amazon Transcribe (ASR-3). According to the context where the conversational system is implemented, where the most of PwADs are elderly people, it is necessary to evaluate the performance of ASR systems in this scenario.

In this regard, a set of eight audio files was assembled. Each audio file was recorded from a series of interviews with 8 people over the age of 65. The questions asked in each interview were the same for all participants and were as follows: (1) what year were you born? (2) where were you born? (3) briefly describe something you like about your birthplace, (4) Can you mention any pleasant memories from your childhood? and (5) briefly describe your normal day’s routine.

From each audio file, the voice of the interviewer asking the questions was removed, preserving only the speech of each participant. Table 3 shows the characteristics of the audio files generated.

The performance of speech recognition is typically measured using word error rate (WER), the ratio of word insertion, substitution, and deletion errors in a transcript to the total number of spoken words [39]. The WER value is obtained by using Equation (1).

(1) $$WER=\frac{S+I+D}{N}$$

where *N* is the total number of words in the transcript, *S* is the number of words that were substituted, *I* is the number of insertions, and *D* is the number of words that were deleted. Accordingly, a value of WER=0 would indicate a perfect transcription, while a high value indicates a low accuracy of the ASR system.

To evaluate the ASR systems, initially a manual transcription of each audio file was made, these transcripts were used as a reference transcript to compare the transcription provided by each system. Subsequently, each audio file was processed by each ASR system and the transcript generated by each ASR system was compared with the reference transcript and thus the WER value was calculated for each file analyzed.

The results obtained from the evaluation of the ASR systems are described in Table 4.

**Figure 5:**
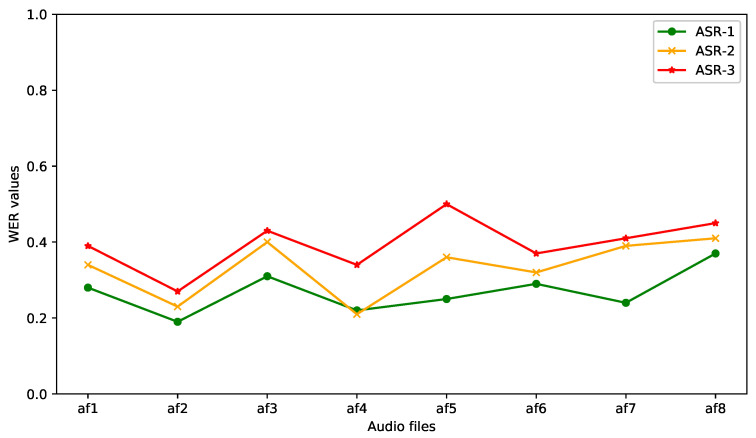
Comparison of the WER values obtained by each ASR system.

Table 4 shows the WER values for each file, as well as the average WER value for each system. It can be observed that the Google Speech-to-Text (ASR-1) system shows better accuracy by having the lowest average WER value (0.268). In Figure 5, it can also be seen that the performance of the ASR-1 system showed the most stable behavior during the processing of the set of audio files.

Some additional observations obtained during the experiment were that the ASR-1 system showed better performance in detecting proper nouns such as names of people and cities, it also recognized more accurately numbers and monetary amounts, and it showed better performance in recognizing food dishes. According to the results obtained it can be determined that the Google Speech-to-Text system obtains the best accuracy compared to the other two systems at least in this particular scenario.

### 5.2. Results of the Conversational System Evaluation

Each dialogue was evaluated by each of the 11 evaluators as was previously mentioned. For each candidate response within these dialogues, the $R_{voted}$ value is calculated—i.e., the number of votes that each utterance (response) obtained when it is considered the most appropriate response based on the dialogue context. In accordance with the number of evaluators, the maximum $R_{voted}$ value for each response could be 11 if the response is selected as appropriated by all the evaluators within a dialogue, and the minimum $R_{voted}$ value is 0 if the response is not selected by any evaluator as appropriate.

**Figure 6:**
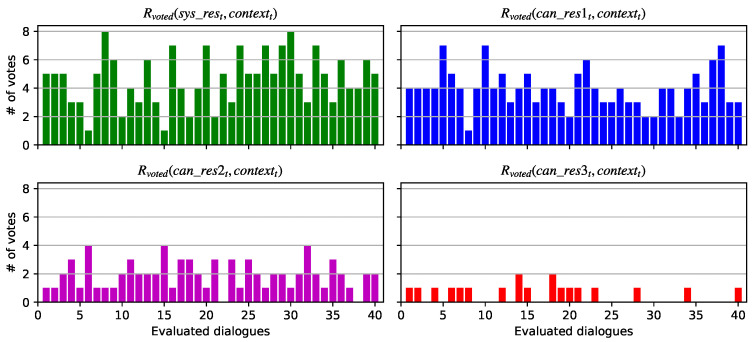
$R_{voted}$ values obtained for each response in the set of evaluated dialogues.

Figure 6 shows the $R_{voted}$ values for each response in each evaluated dialogue. It can be observed that the $R_{voted}\left(res\_sys_{t},context_{t}\right)$ values corresponding to the number of votes obtained by the responses given by the system are higher than the other $R_{voted}$ values in most of dialogues, only followed by the $R_{voted}\left(can\_res1_{t},context_{t}\right)$ corresponding to the candidate response 1. The $R_{voted}$ values for candidate responses 2 and 3 reveal that these were the less voted responses by the evaluators, reaching a maximum of 4 votes and 2 votes in a few dialogues respectively.

This meaning that the responses provided by the conversational system within each dialogue were in most cases the best appropriate response according to the number of votes received by the evaluators.

**Figure 7:**
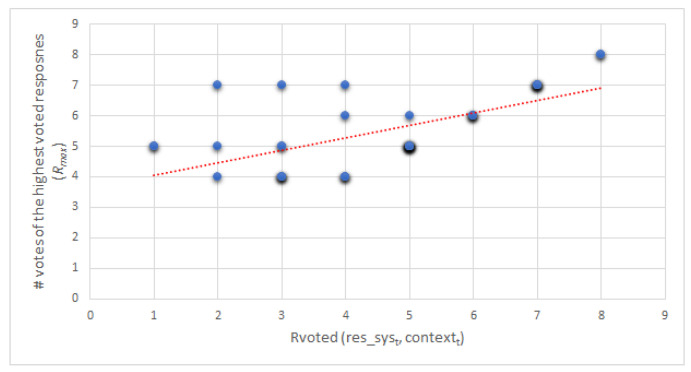
Correlation between $R_{voted}\left(res\_sys_{t},context_{t}\right)$ and $R_{max}$ values. A small amount shadow is added to $R_{voted}$ values for visualization of most cases.

Moreover, once the $R_{voted}$ value was calculated for each response in each of the dialogues, the correlation between the number of votes obtained by the system responses $R_{voted}\left(res\_sys_{t},context_{t}\right)$ and the responses that obtained the highest number of votes in each dialogue ($R_{max}$) was calculated (Figure 7), obtaining a Pearson’s correlation coefficient of 0.634 (p<0.001,n=40). Thus, there is a positive correlation between them. Therefore, it can be observed throughout the evaluation that there is indeed a correlation in that the answer provided by the system is one of the most voted in each dialogue evaluated.

**Figure 8:**
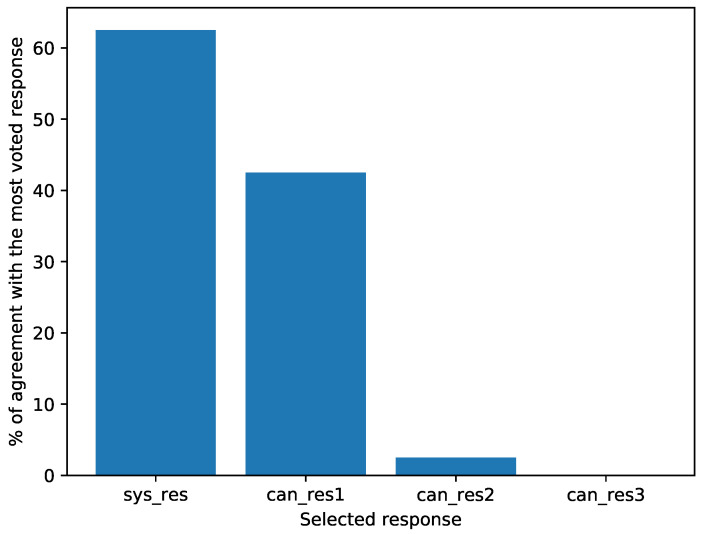
Percentage of agreement between the most voted response and the candidate responses.

In this sense, in Figure 8 can be observed that the responses given by the system obtained the highest percentage (62.5%) as the most voted responses in comparison with the other candidate responses, since candidate responses 1 agreed 42.5% of times with the most voted response, candidate responses 2 only 2.5%, while candidate responses 3 did not agree with the most voted on any occasion.

In addition, at the end of the evaluation of the set of dialogues, the evaluators were asked to give a rating from 1 to 5 stars to system based on the dialogues generated. The majority of the evaluators, 63.6% (n=7) gave 4 stars, 27.3% (n=3) gave 5 stars and 9.1% (n=1) gave 3 stars.

### 5.3. Qualitative Results of the Conversational System

A qualitative evaluation of the system was carried out by means of a survey with two open-ended questions. The objective was to obtain a general evaluation from the participants, in order to get their opinion about the relevance of the system to be used in real patients based on their experience in providing therapy to PwADs. This survey was completed once each evaluator had evaluated the set of dialogues.

The questions addressed to the evaluators (E) were as follows:

- Q1. Do you consider the above dialogues to be appropriate for providing reminiscence therapy?
- Q2. What adjustments could you suggest to improve patient acceptance or interest?

Regarding to Q1, according to their answers the participants mentioned that the responses provided by the system are appropriate. Also, according to their point of view, they mentioned that the use of the system will provide a kind of company to the PwADs so that they will not feel lonely. In this sense, the caregivers agreed that it is necessary for a conversational agent to be perceived as someone with whom patients feel comfortable to talk and interact.

[E2-caregiver]: *“They are suitable because many times we can’t be with our family member talking because we do other things and this way they could talk about their life and not feel alone.”*

[E6-caregiver]: *“I believe they are adequate and give the patient the opportunity to talk and recall.”*

[E9-caregiver]: *“I think that dialogues are good, because it gives the patient the possibility to interact with the caregiver without feeling pressured and also to feel companionship, empathy and support.”*

Regarding to Q2, the participants suggest that the system’s intervention should not only be based on conversations but also based on music, as this is something that patients will enjoy. On the other hand, they also highlight the possibility of the system providing more than one option to follow the conversation with the patient, which can provide better guidance throughout the therapy.

[E5-caregiver]: *“It would be nice if you could add some songs that they could sing.”*

[E7-geriatric doctor]: *“The system responses should be options with the aim to address the reminiscence.”*

The obtained results allow us to affirm that the responses provided by the conversational system are adequate to conduct reminiscence therapy with real PwADs. Therefore, in a next stage of evaluation of the system, we will conduct an intervention with PwADs in a geriatric residence.

## 6. Discussion

The results obtained following the evaluation scheme described above show that although the maximum number of votes that a response could reach in a dialogue scenario is 11, the highest number of votes reached by a response was 8. This is due to the fact that there is a degree of subjectivity in this type of evaluation, since for one person it could be an appropriate response for another person it might not be.

In this sense, although the best way to evaluate the performance of a conversational system is still human judgment, there are many occasions were the answers generated by the system are unlikely to meet with the agreement of all evaluators. Therefore, a good indicator of adequate system performance is usually that the majority of evaluators agree that the system’s responses are the most appropriate according to the dialogue context, as in this case. The results obtained from evaluation task showed that the responses given by the system in different dialogue scenarios are the most appropriated responses based on the number of votes given by the evaluators to each response, reaching the highest number of votes in most dialogues as is showed in Figure 4.

In addition, applying a 5-star rating scheme to evaluate the overall performance of the system, the evaluators gave an average rating of 4.18, which indicates that based on their criteria the system performs well overall in providing therapy to PwADs. Similarly, when conducting the qualitative evaluation of the system, the evaluators also agreed that the dialogues generated by the system are mostly appropriate for providing RT. It is worth noting that even though all evaluators are familiar with the RT and have the experience of having provided it at least once, most of the evaluators are caregivers and not therapists or health specialists. Although this may contribute to the way they evaluate the system, it will be useful to conduct a subsequent evaluation with specialists in RT and compare the differences in the evaluation between these two groups of evaluators. Moreover, it is convenient to increase the number of evaluators, both caregivers and specialists, in order to obtain a more representative evaluation.

Regarding the information gathered from the PwAD, it is important to mention that both the information gathered through the initial form and the information gathered during the interaction with the system is stored in a restricted database and only available for use by the Dialog Manager. The information is associated with each patient’s profile, therefore it cannot be shown or consulted by any other patient or caregiver. If a PwAD’s caregiver or the PwAD himself/herself decides not to continue using the system, his/her information is completely removed from the database.

Finally, because of the production and understanding of language is relatively well preserved in the early stage of Alzheimer’s, proposals that use spoken dialogue systems to support early diagnosis and the development of non-pharmacological treatments have proven to be well adopted by PwADs and caregivers [25,26,28]. Hence, these systems can provide a valuable tool in the treatment of this condition.

### Limitations

There are certain limitations that need to be highlighted. Firstly, concerning the evaluation of the system, at this stage we have only evaluated whether the dialogues generated by the system are suitable for use within the RT. However, it is necessary to evaluate how well the system performs in carrying out the RT, so this evaluation is expected to be carried out at a subsequent stage of this study.

Another limitation is that the system only has the ability to personalize the conversation based on the information collected from the PwADs. Therefore, the system does not perform any external search to obtain information related to the dialogue that could be used to enrich the conversation. In addition, this version of the system can only conduct a RT session through conversation. However, in sessions conducted by therapists, elements that allow reinforcing the RT, such as the use of photographs, music, toys or smells, are commonly used [32]. Therefore, in a future version, we are considering adding the use of photographs and music that could evoke pleasant memories to the patient. Nevertheless, it is important to mention that according to the characteristics of Alzheimer’s disease, the benefits of providing RT cannot be generalized to all PwADs.

## 7. Conclusions and Future Work

There are several proposals focused on providing cognitive therapy and assistive support to patients with dementia. However, few studies are focusing on the development of systems that provide reminiscence therapy and those that do are mostly developed for English language. In this sense, this paper describes a conversational system that can be employed as a tool in providing reminiscence therapy to patients with Alzheimer’s disease in Spanish language, through the generation of personalized conversations related to the life history and interests of each PwAD. This would provide PwADs with the benefits of RT when applied constantly.

Furthermore, relying on the evaluation results of the system, the conversational system proposed in this work has showed that is capable of carrying out a reminiscence therapy according to the PwAD information in a successful manner. However, it is required that the system could be tested and evaluated on interactions with real PwADs. Likewise, as future work we plan carry out the evaluation of the system on real PwADs in a geriatric residence in order to observe and evaluate the interaction between the system and PwADs. The information gathered from this interaction will allow us to enhance the performance of the system in a more realistic context.

In addition, it is proposed to incorporate a module within the architecture of the system that allows the analysis of PwADs utterances and assess the progress of their cognitive impairment by comparing the information they provide during interaction with the system with the information stored in the database. The results obtained of this analysis could be useful to improve or adjust the treatment to which they are subjected.

## Figures and Tables

**Table 1:** Interactive systems focusing on dementia domain.

First Author, Year	Study Purpose	Type of System	Main Findings
Tanka et al., 2017	Dementia diagnosis	Virtual avatar	Achieve an accuracy of 0.93
			in the detection of dementia.
Chinaei et al., 2017	Confusion detection	Dialogue system	Identify confusion from speech
			with up to 82% of accuracy.
Yasuda et al., 2013	Remote reminiscence	Videocall	They observed that psychological
	and daily assistance		stability persisted for 3 hours
			after remote conversation
Rudzicz et al., 2015	Personal assistance	Mobile robot	They found that high levels of
			confusion in PwADs promote that
			ignore the robot prompts (up to 40%)
Wolters et al., 2016	Cognitive assistance	Spoken dialogue system	Voice and interaction style should be
			chosen based on the preferences of
			the user, not those of the caregiver
Casey et al., 2016	Cognitive support	Companion robot	Relevant aspects of acceptance by
			patients are speak slowly and loudly,
			and to know patient’s interests
			and preferences
Navarro et al., 2016	Occupational therapy	Computer application	Personalizing the intervention
			helps to facilitate the adoption
			of this kind of systems
Cruz-Sandoval et al., 2020	Cognitive stimulation	Social robot	They observed a statistically significant
	therapy		decrease in behavioral symptoms

**Table 2:** Dialogue contexts created within the AIML Knowledge Base.

Dialogue Context	Description (Conversations About)
Beginning conversation	welcome greetings to the PwAD
Leave conversation	farewell phrases to the PwAD
Agent profile	information about the conversational system
	(e.g., name, gender, age)
PwAD profile	personal information about the PwAD
	(e.g., birthday, nickname, place of birth)
Family	relevant family members for the PwAD
Habits	daily habits that the PwAD used to perform
Skills	skills in which the PwAD used to be good at
	(e.g., weaving, drawing, singing, woodworking)
Hobbies	preferred hobbies of the PwAD
Household chores	activities at home were the PwAD was happy for helping
Visited places	places visited that were relevant in his life
	PwAD’s preferences about
Movies	movies which has been established as favorites
TV shows	favorite TV shows
Actors	actors and characters of interest to the PwAD
Music	music that is preferred by the PwAD
Singers	singers or bands preferred by the PwAD
Sports	sports that are preferred by the PwAD
Sport teams	teams and athletes relevant to the PwAD
Food dishes	preferred food dishes
Beverages	preferred beverages
Festivities	festivities liked by the PwAD

**Table 3:** Description of the audio files generated.

Audio File	Participant Gender	Age	Audio Duration (min.)
af1	Female	67	3.28
af2	Male	68	5.32
af3	Female	65	4.15
af4	Female	81	4.10
af5	Male	78	6.18
af6	Female	76	3.14
af7	Female	67	5.50
af8	Male	70	4.30

**Table 4:** WER values obtained by the ASR systems.

Audio File	ASR-1	ASR-2	ASR-3
af1	0.28	0.34	0.39
af2	0.19	0.23	0.27
af3	0.31	0.40	0.43
af4	0.22	0.21	0.34
af5	0.25	0.36	0.50
af6	0.29	0.32	0.37
af7	0.24	0.39	0.41
af8	0.37	0.41	0.45
Average	0.268	0.332	0.395

## Abbreviations

The following abbreviations are used in this manuscript:

PwAD	Patient with Alzheimer’s Disease
RT	Reminiscence Therapy
